# Smallpox: An attack scenario.

**DOI:** 10.3201/eid0504.990416

**Published:** 1999

**Authors:** T O'Toole

**Affiliations:** Johns Hopkins School of Public Health, Baltimore, Maryland, USA. biodefen@jhsph.edu


### Smallpox: An Attack Scenario

**Tara O'Toole**
Johns Hopkins School of Public Health, Baltimore, Maryland, USA

Smallpox virus, which is among the most dangerous organisms that might be used by bioterrorists, is not widely available. The international black market trade in weapons of mass destruction is probably the only means of acquiring the virus. Thus, only a terrorist supported by the resources of a rogue state would be able to procure and deploy smallpox. An attack using the virus would involve relatively sophisticated strategies and would deliberately seek to sow public panic, disrupt and discredit official institutions, and shake public confidence in government.

The following scenario is intended to provoke thought and dialogue that might illuminate the uncertainties and challenges of bioterrorism and stimulate review of institutional capacities for rapid communication and coordinated action in the wake of an attack.

### Capacity To Detect a Bioterrorist Attack and To Diagnose an Unusual Disease

April 1

The vice-president visits Northeast, a city of 2.5 million. His itinerary includes an awards ceremony, an appearance at a local magnet school, and a major speech at the local university. A crowd of 1,000 people, including students, is gathered in the university auditorium. Hundreds more wait outside, where the vice-president stops to shake hands and respond to queries from the media.

The Federal Bureau of Investigation (FBI) has information suggesting a possible threat against the vice-president from a terrorist group with suspected links to a rogue state. The group is known to have made inquiries about acquiring biological pathogens, including smallpox, and is suspected of having procured aerosolization equipment. FBI decides its information is too vague and too sensitive to pass on to the Department of Health and Human Services, local law enforcement authorities, or the state health department.

Address for correspondence: Tara O'Toole, Johns Hopkins Center for Civilian Biodefense Studies, Candler Building, Suite 850, 111 Market Place, Baltimore, MD 21202, USA; fax: 410-223-1665; e-mail: biodefen@jhsph.edu.

April 8

FBI informants report rumors that something happened while the vice-president was in Northeast.

April 12

A 20-year-old university student goes to the university hospital emergency room with fever and severe muscle aches. She is pale, has a temperature of 103°F, and is slightly leukopenic, but the physical exam and laboratory results are otherwise normal. She is presumed to have a viral infection and is sent home with instructions to drink fluids and take aspirin or ibuprofen for muscle aches. Later that day, a 40-year-old electrician arrives at the emergency room with severe lower backache, headache, shaking chills, and vomiting. He appears pale and has a temperature of 102°F and a pale erythematous rash on the face. The patient is a native of Puerto Rico, where he visited 10 days earlier. A diagnosis of dengue fever is considered, and the patient is discharged with ibuprofen and instructions to drink fluids.

April 13

Over the course of the day, four young adults in their twenties come to the university hospital emergency room with influenzalike symptoms and are sent home.

April 14

The female student returns to the emergency room after collapsing in class. She now has a red, vesicular rash on the face and arms and appears acutely ill. Her temperature is 102°F; her blood pressure is normal. She is admitted to an isolation room with presumptive diagnosis of adult chickenpox. She has had no contact with others known to have chickenpox.

April 15

The electrician first seen on April 12 returns to the emergency room by ambulance. He too has a vesicular rash and appears very ill. He is also admitted to an isolation room with presumptive diagnosis of chickenpox.

That evening at 6 p.m. the infectious disease consultant and the hospital epidemiologist meet on the elevator. The infectious disease specialist has just finished examining the student and the electrician, both of whom have vesicular rash on the face, arms, hands, and feet. The skin lesions are evolving in phase. The possibility of smallpox is raised. The infectious disease specialist takes a swab specimen from the electrician's skin lesions, sends it to the laboratory, and requests that it be examined by electron microscopy by an experienced technician. The doctor assures the technician that he will be vaccinated if the specimen shows smallpox. At 7:00 p.m., electron microscopy shows an orthopoxvirus consistent with variola—the smallpox virus.

At 7:15 p.m. the hospital epidemiologist declares a contagious disease emergency. The two patients are moved to negative-pressure rooms with HEPA filters. Visitors and hospital staff not already caring for and in contact with patients are forbidden to enter the floor. Infection-control nurses begin interviewing staff to determine who has been in face-to-face contact with the patients during initial emergency room visits and admission. The hospital epidemiologist calls the chair of the department of medicine and the hospital vice-president for medical affairs.

Within 45 minutes the chair of the department of medicine and the president of the hospital are meeting with the infectious disease physician, the hospital epidemiologist, the hospital vice-president for public relations, and the hospital's general counsel. The city and state health commissioners join the meeting by phone. The need to vaccinate and isolate all contacts of the patients is recognized and discussed. It is decided to secure the hospital. No one is allowed to leave until all persons are identified so that they can be vaccinated as soon as vaccine can be obtained from the Centers for Disease Control and Prevention (CDC). The possibility of identifying and vaccinating other patient contacts (e.g., family members not now in the hospital) is discussed, but no decisions are made because the hospital's legal authority for doing this is unclear.

Half an hour later, the state health commissioner calls FBI. He also contacts CDC to request that smallpox vaccine be released for hospital staff and patient contacts. Because vaccine supplies are limited, CDC requests that the diagnosis of smallpox first be confirmed at CDC. CDC calls FBI and arranges to fly a three-person Epidemic Intelligence Service team to Northeast for assistance.

By 9:30 p.m., an FBI special agent arrives at the hospital, secures biological samples taken from the patients, and drives them to Andrews Air Force Base, where a military aircraft flies the samples to CDC's Biosafety Level 4 laboratory in Atlanta, Georgia. FBI requests that city police be called to help maintain order and ensure that no patients, staff, or visitors leave the hospital until all occupants have been identified and their addresses have been recorded. More FBI agents and city police arrive on the hospital grounds.

Hospital visitors are confused and angered by police refusal to allow anyone to leave the hospital. No explanation is given for the containment to staff, visitors, or the police. Ambulances are rerouted to other hospitals. The rumor that smallpox has broken out rapidly spreads through the building, as do rumors that a terrorist wanted by FBI is in the building. A fight erupts between people trying to leave the facility and the police. Three people are injured and sent to the emergency room. More police and FBI agents arrive and surround the building.

The local television networks report the scene outside the hospital on the late night news. The hospital public relations representative explains that the lock-in is temporary and intended only to gather names and addresses so that people can be contacted and treated if a suspected, but unnamed, contagious disease is confirmed. CNN arrives and demands access to the hospital and affected patients. Rumors about what the contagious disease might be include Hong Kong flu, meningitis, Ebola virus, smallpox, and measles.

The mayor and state attorney general's office are contacted by the health commissioner. There is a phone discussion with the hospital's general counsel and epidemiologist about the right to impose quarantine. Visitors, nonessential personnel, and new patients are blocked from entering the hospital, but visitors already in the building are allowed to leave after their names and addresses are recorded.

FBI, however, is reluctant to allow anyone to leave the building. This provokes a lengthy exchange among the FBI agent-in-charge, the city police chief, and hospital administrators and attorneys. The dispute is resolved after a series of phone calls between FBI headquarters and the state attorney general's office.

### Early Response

11:30 p.m.

The specimen arrives at CDC. At midnight, the diagnosis of smallpox is confirmed. A phone conference with hospital staff, the city police chief, the state health commissioner, the state attorney general, the governor, CDC, FBI, an assistant secretary of the Health and Human Service (HHS), and staff from the National Security Council and the White House (32 people in all) focuses on whether and how to release the information to the media. The mayor and the governor will go on television in the morning with the health commissioner. The FBI director will also make a statement. The president will address the country at noon.

CDC makes arrangements to release smallpox vaccine early the next morning for use by patient contacts and the health-care teams caring for hospitalized victims.

April 16

Morning conference calls between CDC, FBI, HHS, the National Security Council, and state health authorities are set up. Federal officials now assume that a bioterrorist attack has occurred in Northeast. There is concern that other attacks might also have taken place but not yet come to light or that further attacks might be imminent.

A representative from the counterterrorism office of the National Security Council asks if it is necessary or desirable to attempt a complete quarantine of Northeast, including closure of the city airport and a ban on rail traffic leaving from or stopping in the city. The group agrees that such a step is neither feasible nor warranted. A heated debate follows about the advisability of vaccinating all hospital staff and visitors at all facilities where a single case of smallpox is clinically suspected. The state health commissioner presses for enough vaccine for the entire city of Northeast.

FBI and CDC are reluctant to begin mass vaccination until the dimensions of the outbreak are better understood. It is decided to vaccinate all hospital staff and any visitors to the floor where the patients were located. All direct contacts of the patients will also be vaccinated. By the end of the long phone conference, the decision is made to vaccinate all health-care personnel, first responders, police, and firefighters in any city with confirmed cases of smallpox.

CDC Epidemic Intelligence Service officers arrive in Northeast to assist the state epidemiologist, who is establishing a statewide surveillance and case investigation system. Efforts begin to develop a registry of all face-to-face contacts of smallpox patients and to monitor, daily, all contacts for fever. Anyone who has fever >101°F is to be isolated, at home if possible, and be followed for rash.

The state health department activates a prearranged phone tree to query all hospitals and walk-in clinics in the state about similar cases and counsels immediate isolation of all suspected patients.

An additional eight admissions for fever and vesicular rash are discovered. All patients are extremely ill; two are delirious. The university hospital emergency room records are searched, and staff attempt to contact all patients who had fever during the previous week. Three more probable smallpox cases are discovered. Telephone follow-up reveals that one has been admitted to another hospital out of state.

CDC and state health officials discuss possible strategies for managing the epidemic if there is insufficient vaccine for all patient contacts, as seems likely. Home isolation of nonvaccinated patient contacts is considered, but the legal authorities, practical logistics, and ethical implications of such a strategy remain unclear and unresolved.

After discussion among state health authorities and university hospital staff, it is decided that the university will serve as the city's smallpox hospital and will accept transfers of smallpox patients now hospitalized at other facilities in the state. Other hospitals will refer patients to the university hospital or to the state armory but will not admit patients with suspected smallpox. Physicians will be urged to avoid seeking admission for most smallpox patients and to care for patients in their homes.

Arrangements are made by the state health commissioner to activate a state disaster plan, which establishes the armory as an emergency hospital for the quarantine of smallpox patients, in case the number of smallpox patients exceeds hospital isolation capabilities.

### Quarantine and Vaccination

During the morning interagency phone conference, Department of Justice representatives raise questions about potential legal liabilities associated with adverse vaccine effects. The questions remain unresolved, but vaccination will proceed.

On the evening of April 16, the president goes on television to inform the nation of the bioterrorist attack by unknown terrorists, vows that the assailants will be identified and brought to justice, and urges calm and cooperation with health authorities.

The initial epidemiologic evidence and FBI information suggest that the smallpox release likely occurred during the vice-president's January speech at the university in Northeast. Efforts are begun to identify and vaccinate everyone who attended the speech. Additional health department personnel are detailed to help in the epidemiologic investigation. Media reports say that the government does not know how many people are sick or how widespread the outbreak might be.

By evening, 35 more cases are identified in eight emergency rooms and clinics around the city; 10 cases are reported in an adjoining state. CDC alerts all state health departments to be on alert for possible smallpox; CDC also urges prompt and strict isolation measures and instructs states to send specimens from suspected patients to its headquarters in Atlanta for definitive laboratory diagnosis.

April 17

In Northeast, 10,000 residents are vaccinated by the city and state health departments with assistance from volunteer physicians and nurses. Vaccination of the entire university student body, faculty, and staff is discussed and rejected by federal officials for fear that vaccine supplies will be needed for contacts of confirmed cases. State health officials continue to press for a statewide vaccination effort. Unions representing nurses and other health-care workers call for vaccination of all employees whose jobs involve direct patient contact.

April 18

An additional 20,000 residents of Northeast are vaccinated.

April 19

CDC and the U.S. Army Medical Research Institute of Infectious Diseases (USAMRIID) determine that the infecting strain of smallpox was not bioengineered. The genomic sequence is entirely typical of known smallpox strains.

The student with the first diagnosed case dies. Ten more smallpox cases have been identified, bringing the number of confirmed cases to 50. The patients are located in four states, all in the mid-Atlantic area. Suspected cases are identified in five other states.

April 20

Governors of affected and unaffected states press, both behind the scenes and publicly, for emergency vaccine stocks to be distributed to states so that immediate action can be taken should an outbreak occur.

At the close of day 4 of the vaccination campaign, 80,000 have been vaccinated.

April 22-27

No new cases of smallpox with onset after April 19 have been confirmed, although many suspected cases with fever and rash due to other causes are being seen. In the states reporting confirmed smallpox cases, thousands of people are seeking medical care because of worrisome symptoms. CDC and state health authorities decide to issue a recommendation that patients with fever who cannot be definitively diagnosed be strictly quarantined and observed until the fever subsides. CDC and state health departments are flooded with calls from health-care providers seeking guidance on isolation procedures.

Some hospitals and health maintenance organizations (HMOs) complain to HHS that they cannot afford to isolate the many patients with fever and rash at their facilities and demand that the government pay quarantine costs. State health departments are similarly worried about the costs of quarantine.

Local media report an outbreak of sick children with rash in an area elementary school.

It is unclear whether the illness is chickenpox or smallpox. Television stations show film of parents arriving at school in midday to remove children from classrooms. A college basketball star is rushed to hospital by ambulance with an unknown illness. Local television reports that the athlete has high fever but no rash. Both stories are covered on the national evening news.

April 28

Smallpox is diagnosed in two young children in Megalopolis, a large city in another state. FBI and the National Security Council worry that these cases might signal another attack since the children have had no discernible contact with a smallpox patient or contacts. The possibility that there has been a new attack is weighed against the possibility that the children were infected by a contact of one of the first wave of patients who was missed in the epidemiologic investigation.

Members of the state congressional delegation demand that the federal government implement a massive citywide vaccination program. CDC notes that a Megalopolis-wide vaccination program would deplete the entire civilian vaccine supply.

The media report that the president, vice-president, cabinet representatives, and prominent members of Congress have been vaccinated, and the military has already begun to vaccinate the troops in affected states and Washington, D.C.

### The Epidemic Expands

April 29

Over the course of the day, CDC receives reports of an additional 100 new cases of potential smallpox. Sixty of these are in the original state. The others are scattered over eight states. It is not immediately clear if these are truly smallpox or mistaken diagnoses. By evening, laboratory confirmation of smallpox is obtained at CDC. Two cases in Montreal and one in London are also reported. CDC and health agencies now recognize that they are seeing a second generation of smallpox cases. It is presumed that the latest victims were infected by contact with those who attended the vice-president's speech, but a second bioterrorism attack cannot be immediately ruled out. CDC enlists additional epidemiologists from around the country to join teams tracking patients and their contacts.

Another 200 probable cases are reported during the day. CDC receives thousands of requests for vaccine from individual physicians and announces that vaccine will be distributed only through state health departments. Governors of a dozen states are calling the White House, demanding vaccine. One state attorney general announces a suit against the federal government to force release of vaccine for a large-scale vaccination campaign.

The federal government announces that 90% of available vaccine stocks will be distributed to affected states, but cautions that the available quantity of vaccine can cover only 15% of those states' populations. Governors are to determine their own state-specific priorities and mechanisms of vaccine distribution. Federal officials also announce an accelerated crash vaccine-production program that will reduce vaccine-manufacturing time to 24 months.

April 30

A well-known college athlete dies of hemorrhagic smallpox. The rumor is reported that he was the victim of a new biological attack using a different organism since he did not develop the rash associated with classic smallpox. Television commentators misinterpret technical statements from a health-care expert; the commentators report that the athlete died of hemorrhagic fever, and they read clinical descriptions of Ebola virus infection on the air.

The White House and CDC receive dozens of calls from furious governors, mayors, and health commissioners, demanding to know why they were not informed of additional bioterrorist attacks using Ebola. Nurses, doctors, and hospital-support personnel in health centers walk off the job. Thousands of people who attended college basketball games where the deceased athlete played call the health department and ask for treatment.

HHS issues a press release explaining that the athlete did not have Ebola virus. FBI affirms that there is no reason to believe that an attack using any hemorrhagic fever virus has occurred, but FBI refuses to rule out the possibility that there has been more than a single bioterrorist attack using smallpox.

April 31

The widely publicized death of the college basketball star, plus dramatic footage of young children covered with pox, drive thousands of people to emergency rooms and doctors' offices with requests for vaccination and evaluation of fever and other symptoms. This escalation in requests for evaluation and care hampers the ability of state health authorities and CDC to confirm the number of actual new cases.

May 1

The number of smallpox cases continues to grow. There are now >700 reported cases worldwide. In Northeast, the capacity of local hospitals to accommodate patients needing isolation has long been exceeded. Smallpox cases and suspected contacts are being isolated in the local armory and convention center, where volunteer physicians and nurses are providing care.

May 5

Epidemiologists are working around the clock to interview patients, trace the chain of infection, place contacts under surveillance, and isolate smallpox victims. The evidence continues to indicate that the vice-president's visit to Northeast was the occasion for the release, but some authorities remain concerned about multiple releases.

May 15-29

The third generation of the epidemic begins. Cases are reported in Northeast, parts of the country far beyond Northeast, and worldwide. The death rate remains 30%. Vaccine supplies are exhausted. Public concern is mounting rapidly. The president has declared states with the largest numbers of victims and people in quarantine to be disaster areas. Congress votes to release federal funds to pay for costs of quarantine. Over the next 2 weeks, 7,000 cases will have been reported.

May 30

The fourth generation of cases begins. By mid-June, 15,000 cases of smallpox will be reported in the United States. Twenty states report cases, as do four foreign countries. More than 2,000 will have died. The deceased include two members of the vice-president's staff and a secret service agent.

The city of Northeast, which is hardest hit by the epidemic, has experienced several outbreaks of civil unrest. The National Guard has been called in to help police keep order and to guard the facilities where smallpox cases and contacts are isolated. The mayor of Northeast is hospitalized with a heart attack.

### Conclusions

The rate of development of new smallpox cases reported worldwide now appears to be stabilizing and perhaps subsiding. Vaccination of contacts has undoubtedly been of benefit. Perhaps more important is the seasonal decrease in the spread of virus as warmer weather returns.

Many business conventions scheduled to convene in Northeast during the early summer are canceled. Tourist trade, a major source of state income, is at a standstill. Many small businesses in the city have failed because suppliers and customers are reluctant to visit the area. Attendance at theaters and sports events is down markedly. In several states, public schools are dismissed 1 month early, in part because parents, fearful of contagion, are keeping their children home, and partly because teachers are refusing to come to work. Across the country, people refuse to serve on juries or attend public meetings for fear of contracting smallpox. In hospitals and HMOs where staff have not been vaccinated, health-care personnel have staged protests, and some have walked off the job.

The exponential increase in cases around the globe has caused some governments to institute strict, harshly enforced isolation and quarantine procedures. Human rights organizations report numerous cases of smallpox patients being abandoned to die or of recovering patients being denied housing and food.

Domestic and international travel is greatly reduced. Travelers avoid countries known to have smallpox. Some countries refuse to admit U.S. citizens without proof of recent smallpox vaccination. Others have imposed 14-day quarantines on all persons entering the country from abroad. A lucrative black market in falsified vaccination certificates has sprung up.

Congress has begun oversight investigations into the epidemic. A congressman accuses the U.S. Food and Drug Administration of deliberately obstructing the development of smallpox vaccine and vows to hold hearings into the matter. Congressional investigations of what FBI knew, when they knew it, and whom they talked with, are ongoing. Multiple lawsuits have been filed on behalf of and against HMOs, hospitals, and state and federal governments. Several large HMOs refuse to pay states for costs associated with caring for patients in isolation wards and quarantine facilities. The states with largest numbers of cases have spent millions of dollars on the epidemic, including establishing quarantine operations, paying for added public health personnel, and overtime pay for police.

In the United States, periodic rumors of miracle treatments, many fueled by the media, provoke ardent demands on a beleaguered health-care system. Since vaccine supplies were depleted, many people seeking protection have turned to ancient techniques. Some physicians are practicing arm-to-arm transfer of vaccinia, with a few attempting immunization with inoculation of smallpox virus from pustules.

Smallpox continues to spread in many parts of the world, echoing its formerly endemic character. Without vaccine, the only control method is isolation, which hinders, but cannot halt, the spread of the disease. By year's end, endemic smallpox is reestablished in 14 countries. The World Health Assembly schedules a debate on reenacting a global smallpox eradication campaign.

Dr. O'Toole is a senior fellow at the Johns Hopkins University Center for Civilian Biodefense Studies. The Center, sponsored by the Hopkins Schools of Public Health and Medicine, is dedicated to informing policy decisions and promoting practices that would help prevent the use of biological weapons.

